# Updated Meta-Analysis on Vitamin Supplementation for Chronic Pruritus: Expanding Evidence Beyond Vitamin D

**DOI:** 10.3390/ijms26083840

**Published:** 2025-04-18

**Authors:** Wu-Hsien Kuo, Ko-Shih Chang, Mu-Hsin Chang, Yao Hsiao, Ru-Yin Tsai

**Affiliations:** 1 Department of Gastroenterology and Hepatology, Yuan Sheng-Branch, Yuan-Rung Hospital, Changhua 51052, Taiwan; wuhsienku@gmail.com; 2 Department of Cardiology, Yuan Sheng-Branch, Yuan-Rung Hospital, Changhua 51052, Taiwan; yrh2702@gmail.com; 3 Division of Cardiovascular Medicine, Yuan Rung Hospital, Changhua 51052, Taiwan; muhsin.chang@msa.hinet.net; 4 School of Medicine, Chung Shan Medical University, Taichung 40201, Taiwan; s1001085@gm.csmu.edu.tw; 5 Department of Anatomy, School of Medicine, Chung Shan Medical University, Taichung 40201, Taiwan; 6 Department of Medical Education, Chung Shan Medical University Hospital, Taichung 40201, Taiwan

**Keywords:** inflammation, vitamin, health prevention, chronic non-communicable diseases, niacinamide, cytokine

## Abstract

Chronic pruritus is a distressing condition associated with various dermatological and systemic diseases, significantly impairing patients’ quality of life. While conventional treatments such as antihistamines and corticosteroids offer relief, their efficacy varies, and long-term use may lead to adverse effects. Emerging evidence suggests that certain vitamins, including vitamin D, vitamin E, vitamin B12, and niacinamide (B3), may play a role in alleviating pruritus through their anti-inflammatory, immune-regulatory, and skin barrier-enhancing properties. However, the effectiveness of these vitamins in managing chronic pruritus remains unclear. This meta-analysis aims to update and expand the evaluation of vitamin supplementation in reducing pruritus severity across different underlying conditions, extending the scope beyond vitamin D to include vitamins B and E. A comprehensive search was performed across PubMed, Embase, Web of Science, and Cochrane Library databases up to January 2025 to identify randomized controlled trials (RCTs) evaluating the effects of vitamin supplementation on chronic pruritus. A total of 21 RCTs (*n* = 1723) were included in the meta-analysis. Compared to placebo, vitamin supplementation demonstrated a significant reduction in pruritus severity (Standardized Mean Difference [SMD]: −0.578, 95% CI: −0.736 to −0.419, *p* = 0.000; *I*^2^ = 53.630, *p* = 0.003). Subgroup analysis revealed that topical vitamin B12 and vitamin D3 showed the most pronounced antipruritic effects, particularly in patients with atopic dermatitis and chronic kidney disease-associated pruritus. Sensitivity analysis confirmed the robustness of the findings; however, potential publication bias was suggested by Egger’s regression test (*p* = 0.00979), indicating that the overall effect may be influenced by small-study effects or underreporting of negative results. This meta-analysis indicates that vitamin B, D, and E supplementation may serve as effective adjunct therapies for managing chronic pruritus. However, the variability among the included studies highlights the necessity for well-structured, long-term RCTs to determine the ideal dosage, treatment duration, and target patient populations that would derive the greatest benefit from vitamin-based interventions.

## 1. Introduction

Chronic pruritus is a distressing and often intractable symptom that significantly impairs quality of life and is associated with a range of dermatological and systemic disorders, including atopic dermatitis, psoriasis, chronic kidney disease (CKD), cholestatic liver disease, and neuropathic conditions [1,2,3]. While antihistamines, corticosteroids, and immunosuppressants remain the mainstay of treatment, their efficacy is often limited, and long-term use is associated with adverse effects [4,5]. Given these limitations, there is growing interest in alternative and adjunctive therapies, including micronutrient-based interventions.

Recent studies suggest that vitamins play an essential role in modulating pruritus through diverse mechanisms, including immune regulation, skin barrier enhancement, anti-inflammatory activity, and oxidative stress reduction [6,7]. Among them, vitamin D [8,9], vitamin E [10], vitamin B12 [11], and niacinamide (B3) [12] have been explored for their potential antipruritic effects in different conditions. Vitamin D is well-recognized for its immunomodulatory properties and has been implicated in reducing inflammatory responses linked to pruritus in conditions such as atopic dermatitis and CKD-associated pruritus [13,14]. Vitamin E, a potent antioxidant, may alleviate pruritus by reducing oxidative stress and stabilizing cell membranes, particularly in inflammatory skin diseases [15]. Vitamin B12 has demonstrated efficacy as a topical treatment for atopic dermatitis, potentially by inhibiting nitric oxide synthase and thereby mitigating neurogenic inflammation [16]. Similarly, niacinamide, known for its barrier-protective and anti-inflammatory properties, has been investigated for its role in reducing pruritus, especially in barrier-compromised conditions [17].

Despite these promising findings, the efficacy of vitamins in managing chronic pruritus remains inconclusive, as individual studies report varying results depending on the underlying disease, intervention duration, dosage, and mode of administration. To address these gaps, we conducted an updated meta-analysis to systematically evaluate the role of multivitamin approaches in chronic pruritus management. This study synthesizes evidence from randomized controlled trials (RCTs) to assess whether vitamin supplementation is an effective strategy for alleviating pruritus across different dermatological and systemic conditions. The findings of this analysis aim to provide clinically relevant insights into the therapeutic potential of vitamins in dermatology, guiding future research and treatment strategies for chronic pruritus.

## 2. Results

### 2.1. Study Search and Characteristics of Included Patients

**Figure 1:**
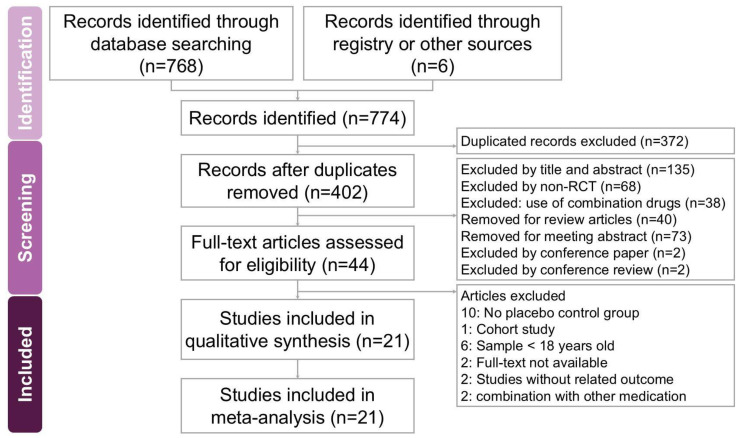
A flowchart depicting the study selection process for the systematic review and meta-analysis on the effects of vitamin supplementation in managing chronic pruritus across various dermatological conditions. Out of 774 initially identified records, 21 studies met the eligibility criteria and were included in the final analysis.

The search and selection process for trials yielded a comprehensive set of studies for inclusion in this meta-analysis (Figure 1). Our initial search across four databases (PubMed, Embase, Cochrane Library, and Web of Science) along with an additional search using PubMed’s ‘related articles’ feature, yielded a total of 774 trials. After removing duplicates, 402 unique trials remained and were subjected to title and abstract screening, resulting in the exclusion of 358 trials. A detailed full-text review of the 44 remaining trials led to the exclusion of 23 trials for reasons including the absence of a placebo control group (10 trials [18,19,20,21,22,23,24,25,26,27]), being a cohort study (1 trial [28]), involving participants under 18 years old (6 trials [29,30,31,32,33,34]), lack of full-text availability (2 trials [35,36]), combination with other medication (2 trial [37,38]) and outcomes unrelated to the study’s focus (2 trials [39,40]). Ultimately, 21 trials met the inclusion criteria and were included in this meta-analysis [7,8,9,10,11,12,41,42,43,44,45,46,47,48,49,50,51,52,53,54,55]. Table 1 summarizes the key characteristics of the included trials, which were published between 2004 and 2023. These 21 trials involved a total of 1723 participants, with the number of participants per trial ranging from 11 to 273. The primary objective of all included trials was to assess the potential effects of vitamin D on chronic pruritus.

### 2.2. Quality Assessment

**Figure 2:**
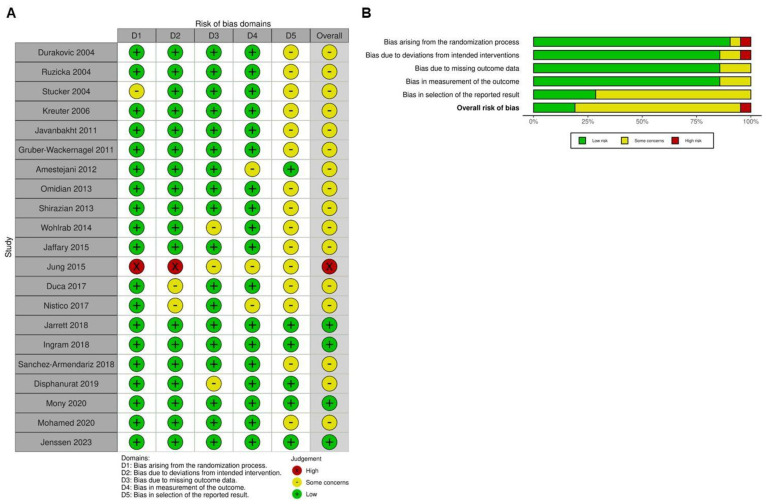
Evaluation of the methodological quality of the included trials. (**A**) Individual risk of bias assessment for each selected study, based on the Rob 2.0 tool (https://mcguinlu.shinyapps.io/robvis/). (**B**) Overall risk of bias summarized as a percentage, considering intention-to-treat and per-protocol analyses. The primary sources of high risk of bias across the studies were deviations from intended interventions, followed by issues related to missing outcome data and deficiencies in the randomization process [7,8,9,10,11,12,41,42,43,44,45,46,47,48,49,50,51,52,53,54,55].

Most of the included trials (Figure 2) were identified as having some concerns of bias, particularly in the selection of reported outcomes. Most studies exhibited a low risk of bias in the randomization process, suggesting that randomization was generally well-executed. However, certain studies, such as Stücker (2004; [7]) and Jung (2015; [9]), lacked sufficient details, resulting in uncertainty in this domain. In terms of deviations from intended interventions, most trials maintained a low risk of bias, indicating that the interventions were administered as planned without substantial deviations that could influence the results. Nevertheless, Jung (2015; [9]) demonstrated a higher risk of bias in this area, which may have impacted its findings. Regarding missing outcome data, the risk of bias varied across studies, with Wohlrab (2014; [12]), Jung (2015; [9]), and Disphanurat (2019; [53]) exhibiting some concerns due to incomplete data reporting. In the assessment of outcome measurement, most trials were considered to have a low risk of bias, but Amestejani (2012; [46]), Jung (2015; [9]), and Nistico (2017; [52]) were flagged as having some concerns, likely due to their open-label study designs. Overall, studies such as Jung (2015; [9]) demonstrated a high risk of bias across multiple domains, which may affect the reliability of their findings. Conversely, other studies, including [49,51,54,55], were categorized as having a low overall risk of bias, suggesting that their results are more reliable and robust. While most studies exhibited a low risk of bias in key areas, some showed high or unclear risks across multiple domains, necessitating careful interpretation of their findings in the broader context of this meta-analysis.

### 2.3. Impact of Vitamin Supplementation on Chronic Pruritus

**Figure 3:**
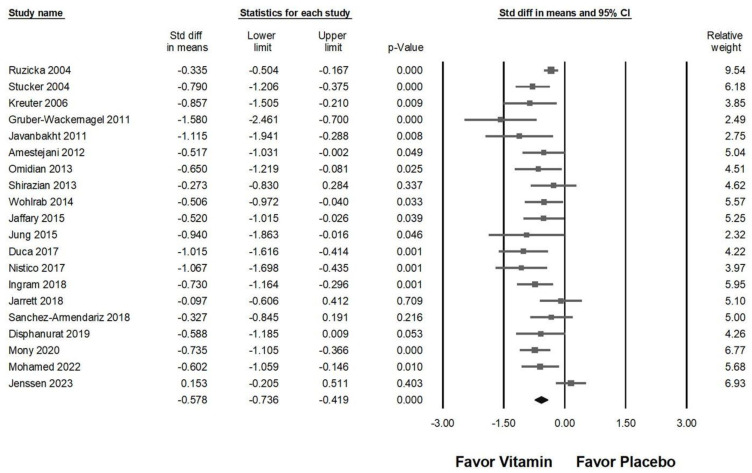
Displays the overall impact of vitamins on chronic pruritus, as measured by the visual analog scale, compared to placebo [7,8,9,10,11,12,42,43,44,45,46,47,48,49,50,51,52,53,54,55].

**Figure 4:**
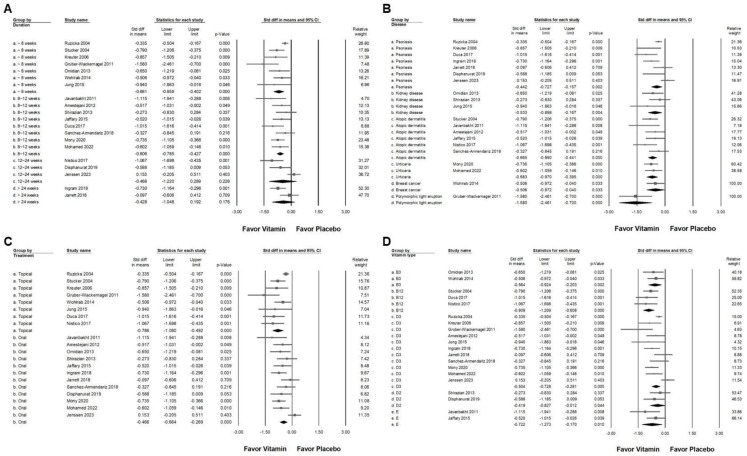
A forest plot illustrating the subgroup analyses of vitamin supplementation in managing chronic pruritus. (**A**) Effect of treatment duration, (**B**) disease, (**C**) route of administration, and (**D**) different vitamin types. The squares represent the effect sizes, with horizontal lines indicating the 95% confidence intervals. The diamond symbol at the bottom of each panel summarizes the overall effect size.

As illustrated in Figure 3, the intervention demonstrated a moderate effect in alleviating pruritus among affected patients (overall effect: −0.578, 95% CI: −0.736 to −0.419, *p* < 0.001; *I*^2^ = 53.630%, *p* = 0.003). Subgroup analysis further indicated a significant reduction in pruritus for those receiving vitamin supplementation compared to placebo (Figure 4A), particularly in cases where the intervention lasted less than 8 weeks (overall effect: −0.681, 95% CI: −0.959 to −0.402, *p* < 0.001; *I*^2^ = 55.737%, *p* = 0.035). In contrast, patients receiving supplementation for 8 to 12 weeks experienced a moderate effect (overall effect: −0.606, 95% CI: −0.785 to −0.427, *p* < 0.001; *I*^2^ < 0.001%, *p* = 0.491). Interventions lasting between 12 and 24 weeks did not demonstrate a statistically significant reduction in pruritus (overall effect: −0.466, 95% CI: −1.220 to 0.289, *p* = 0.226) and were associated with substantial heterogeneity (*I*^2^ = 84.007%, *p* = 0.002). Similarly, interventions exceeding 24 weeks showed no significant effect (overall effect: −0.428, 95% CI: −1.048 to 0.192, *p* = 0.176), with moderate heterogeneity (*I*^2^ = 70.980%, *p* = 0.063). These results imply that prolonged durations of vitamin supplementation do not necessarily yield greater antipruritic benefits.

### 2.4. Subgroup Analysis of Vitamin Supplementation Effects on Chronic Pruritus

In the subgroup analysis based on disease diagnosis (Figure 4B), vitamin supplementation demonstrated a small effect in reducing pruritus among patients with psoriasis (overall effect: −0.442, 95% CI: −0.727 to −0.157, *p* = 0.002; *I*^2^ = 68.673%, *p* = 0.004). A moderate effect was observed in individuals with chronic kidney disease (overall effect: −0.533, 95% CI: −0.898 to −0.167, *p* = 0.004; *I*^2^ < 0.001%, *p* = 0.418), atopic dermatitis (overall effect: −0.665, 95% CI: −0.890 to −0.441, *p* < 0.001; *I*^2^ = 6.109%, *p* = 0.377), urticaria (overall effect: −0.683, 95% CI: −0.970 to −0.395, *p* < 0.001; *I*^2^ < 0.001%, *p* = 0.658), and breast cancer-associated pruritus (overall effect: −0.506, 95% CI: −0.972 to −0.040, *p* = 0.033; *I*^2^ < 0.001%, *p* > 0.999). Notably, polymorphic light eruption exhibited the most significant response to vitamin supplementation, with a large effect observed (SMD: −1.580, 95% CI: −2.461 to −0.700, *p* < 0.001; *I*^2^ < 0.001%, *p* > 0.999). The effectiveness of vitamin supplementation varied based on the route of administration (Figure 4C). The topical application resulted in a significant reduction in pruritus compared to placebo (overall effect: −0.786, 95% CI: −1.080 to −0.492, *p* < 0.001; *I*^2^ = 63.314%, *p* = 0.008). Conversely, oral supplementation exhibited a small effect (overall effect: −0.466, 95% CI: −0.664 to −0.268, *p* < 0.001; *I*^2^ = 47.494%, *p* = 0.034). Among the various vitamin types (Figure 4D), vitamin D2 showed a small effect (overall effect: −0.419, 95% CI: −0.827 to −0.012, *p* = 0.044; *I*^2^ < 0.001%, *p* = 0.449). In contrast, moderate effects were observed in patients receiving vitamin B3 (overall effect: −0.564, 95% CI: −0.924 to −0.203, *p* = 0.002; *I*^2^ < 0.001%, *p* = 0.702), vitamin D3 (overall effect: −0.504, 95% CI: −0.728 to −0.281, *p* < 0.001; *I*^2^ = 63.731%, *p* = 0.002), and vitamin E (overall effect: −0.722, 95% CI: −1.273 to −0.170, *p* = 0.010; *I*^2^ = 31.703%, *p* = 0.226). Vitamin B12 supplementation demonstrated the greatest effect in reducing pruritus, with a large effect size (overall effect: −0.909, 95% CI: −1.209 to −0.608, *p* < 0.001; *I*^2^ < 0.001%, *p* = 0.714). The findings suggest that the efficacy of vitamin supplementation in reducing pruritus varies depending on the disease type, mode of administration, and specific vitamin used.

### 2.5. Effectiveness of Different Vitamin Types and Administration Routes in Pruritus Management

**Figure 5:**
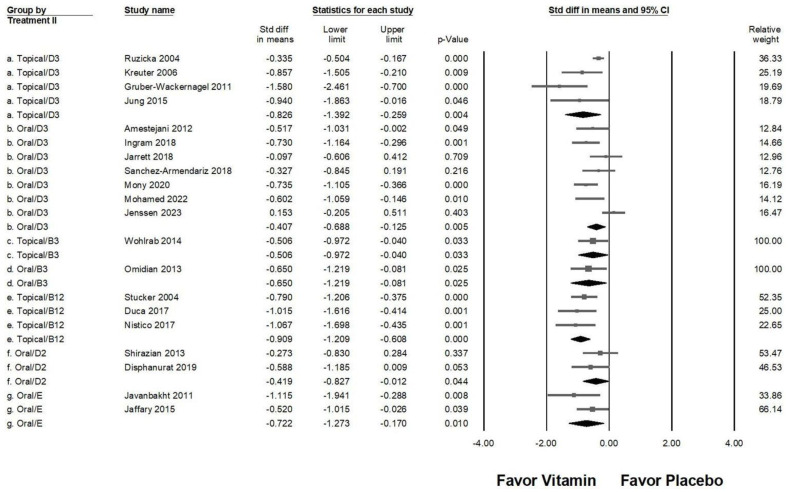
A forest plot illustrating the combined effects of vitamin supplementation on chronic pruritus, integrating both the route of administration and vitamin type. The squares represent individual study effect sizes, with horizontal lines indicating 95% confidence intervals, while the diamond symbol at the bottom of each panel represents the overall pooled effect size.

In the advanced subgroup analysis examining different vitamin types and administration routes (Figure 5), topical vitamin D3 (overall effect: −0.826, 95% CI: −1.392 to −0.259, *p* = 0.004; *I*^2^ = 71.683%, *p* = 0.014) and topical vitamin B12 (overall effect: −0.909, 95% CI: −1.209 to −0.608, *p* < 0.001; *I*^2^ <0.001%, *p* = 0.714) exhibited the strongest antipruritic effects. Moderate pruritus reduction was observed with topical vitamin B3 (overall effect: −0.506, 95% CI: −0.972 to −0.040, *p* = 0.033; *I*^2^ < 0.001%, *p* > 0.999), oral vitamin B3 (overall effect: −0.650, 95% CI: −1.219 to −0.081, *p* = 0.025; *I*^2^ < 0.001%, *p* > 0.999), and oral vitamin E (overall effect: −0.722, 95% CI: −1.273 to −0.170, *p* = 0.010; *I*^2^ = 31.703%, *p* = 0.226). On the other hand, oral vitamin D2 (overall effect: −0.419, 95% CI: −0.827 to −0.012, *p* = 0.044; *I*^2^ < 0.001%, *p* = 0.449) and oral vitamin D3 (overall effect: −0.407, 95% CI: −0.688 to −0.125, *p* = 0.005; *I*^2^ = 64.537%, *p* = 0.010) were associated with a small yet significant reduction in pruritus. These findings suggest that topical applications of vitamin D3 and B12 may be the most effective in alleviating pruritus, while oral vitamin B3, B12, and E also provide considerable benefits. Conversely, oral vitamins D2 and D3, though effective, demonstrated a comparatively weaker impact on pruritus severity.

### 2.6. Comparative Efficacy of Vitamin Types, Administration Methods, and Treatment Duration in Pruritus Management

**Figure 6:**
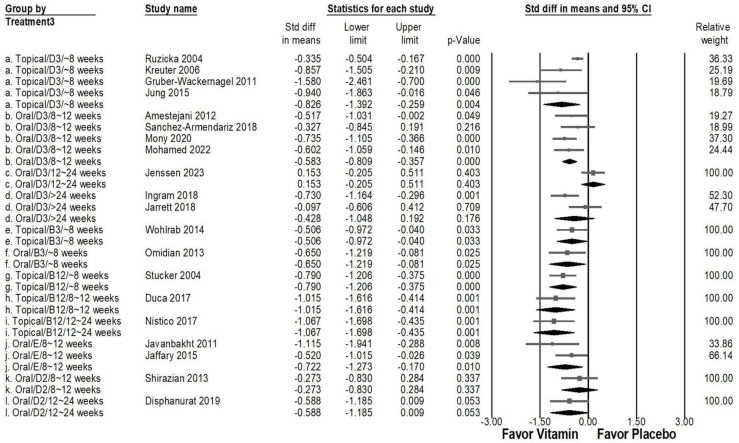
A forest plot depicting the combined effects of vitamin supplementation on chronic pruritus, considering administration route, treatment duration, and vitamin type. The squares represent effect sizes of individual studies, with horizontal lines indicating 95% confidence intervals, while the diamond symbol at the bottom of each panel represents the overall pooled effect size.

In an in-depth subgroup analysis assessing the effects of various vitamin types, administration routes, and treatment durations (Figure 6), topical vitamin B12 applied for 8–12 weeks (overall effect: −1.015, 95% CI: −1.616 to −0.414, *p* = 0.001; *I*^2^ < 0.001%, *p* > 0.999) and 12–24 weeks (overall effect: −1.067, 95% CI: −1.698 to −0.435, *p* = 0.001; *I*^2^ < 0.001%, *p* > 0.999) exhibited a strong antipruritic effect. Likewise, topical vitamin D3 used for less than 8 weeks (overall effect: −0.826, 95% CI: −1.392 to −0.259, *p* = 0.004; *I*^2^ = 71.683%, *p* = 0.014) resulted in a significant reduction in pruritus severity. Moderate effects were observed with oral vitamin D3 for 8–12 weeks (overall effect: −0.583, 95% CI: −0.809 to −0.357, *p* < 0.001; *I*^2^ < 0.001%, *p* = 0.645), oral vitamin B3 for less than 8 weeks (overall effect: −0.650, 95% CI: −1.219 to −0.081, *p* < 0.025; *I*^2^ < 0.001%, *p* > 0.999), oral vitamin E for 8–12 weeks (overall effect: −0.722, 95% CI: −1.273 to −0.170, *p* = 0.010; *I*^2^ = 31.703%, *p* = 0.226). Additionally, moderate effects were noted for topical vitamin B3 applied for less than 8 weeks (overall effect: −0.506, 95% CI: −0.972 to −0.040, *p* = 0.033; *I*^2^ < 0.001%, *p* > 0.999) and topical vitamin B12 administered for under 8 weeks (overall effect: −0.790, 95% CI: −1.206 to −0.375, *p* < 0.001; *I*^2^ < 0.001%, *p* > 0.999). Conversely, oral vitamin D3 administered for 12–24 weeks (overall effect: −0.153, 95% CI: −0.205 to 0.511, *p* = 0.403; *I*^2^ < 0.001%, *p* > 0.999), oral vitamin D3 was taken for up to 24 weeks (overall effect: −0.428, 95% CI: −1.048 to 0.192, *p* = 0.176; *I*^2^ = 70.980%, *p* = 0.063), oral vitamin D2 used for 8–12 weeks (overall effect: −0.273, 95% CI: −0.830 to 0.284, *p* = 0.337; *I*^2^ < 0.001%, *p* > 0.999), and oral vitamin D2 for 12–24 weeks (overall effect: −0.588, 95% CI: −1.185 to −0.009, *p* = 0.053; *I*^2^ < 0.001%, *p* > 0.999) demonstrated a non-significant effect in reducing pruritus.

### 2.7. Impact of Vitamin Supplementation on Skin Lesion Reduction and Inflammatory Cytokine Suppression

**Figure 7:**
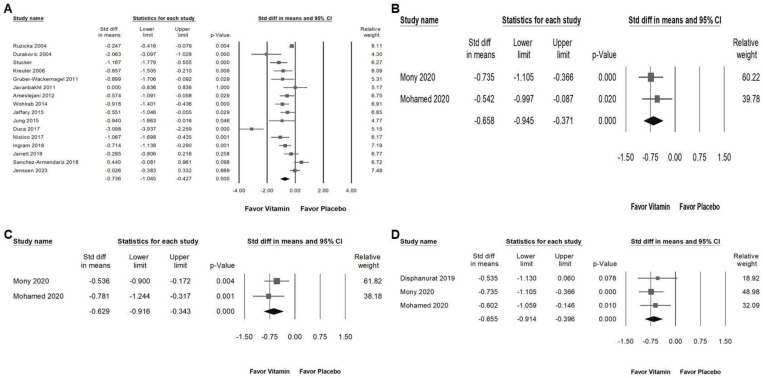
Presents a forest plot that highlights the effects of vitamin supplementation. The plot is divided into four sections for ease of interpretation: (**A**) shows the effect on skin lesion area, (**B**) depicts changes in TNF levels, (**C**) assesses alterations in IL-6 levels, and (**D**) evaluates the impact on hs-CRP. The horizontal lines extending from the squares represent the 95% confidence intervals, while the diamond symbols indicate the overall effect sizes.

Vitamin supplementation significantly reduced skin lesion area, as shown in Figure 7A (overall effect: −0.736, 95% CI: −1.045 to −0.427, *p* < 0.001; *I*^2^ = 83.230%, *p* < 0.001). Furthermore, vitamins exhibited a moderate inhibitory effect on inflammatory cytokines, including TNF-α (Figure 7B; overall effect: −0.658, 95% CI: −0.945 to −0.371, *p* < 0.001; *I*^2^ < 0.001%, *p* = 0.518), IL-6 (Figure 7C; overall effect: −0.629, 95% CI: −0.916 to −0.343, *p* < 0.001; *I*^2^ < 0.001%, *p* = 0.416), and hs-CRP (Figure 7D; overall effect: −0.655, 95% CI: −0.914 to −0.396, *p* < 0.001; *I*^2^ < 0.001%, *p* = 0.823). These findings suggest that vitamin supplementation may play a role in both reducing lesion severity and modulating inflammatory responses in individuals with chronic pruritus.

### 2.8. Sensitivity Analysis

**Figure 8:**
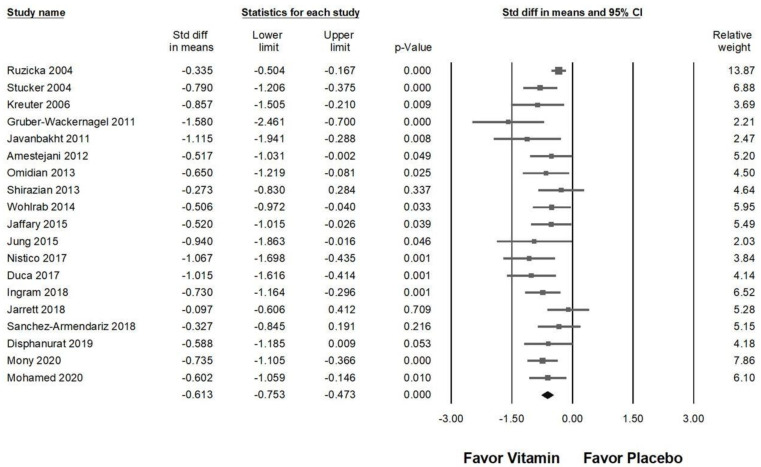
A sensitivity analysis evaluating the effects of vitamin supplementation on chronic pruritus, measured by the visual analog scale, compared to placebo following the exclusion of Jenssen (2023) [54]. The horizontal lines extending from the squares represent the 95% confidence intervals, while the diamond symbols indicate the overall effect sizes.

Among the 20 studies included in Figure 3, one trial that investigated vitamin D3 [54] did not demonstrate a significant effect in relieving chronic pruritus. As a result, this study was excluded from the sensitivity analysis. Figure 8 illustrates the impact of vitamin supplementation on pruritus reduction (overall effect: −0.613, 95% CI: −0.753 to −0.473, *p* < 0.001; *I*^2^ = 34.509%, *p* = 0.070). The results of the sensitivity analysis remained consistent with the initial findings, reinforcing that vitamin supplementation maintains a moderate effect in alleviating chronic pruritus.

### 2.9. Publishing Bias

**Figure 9:**
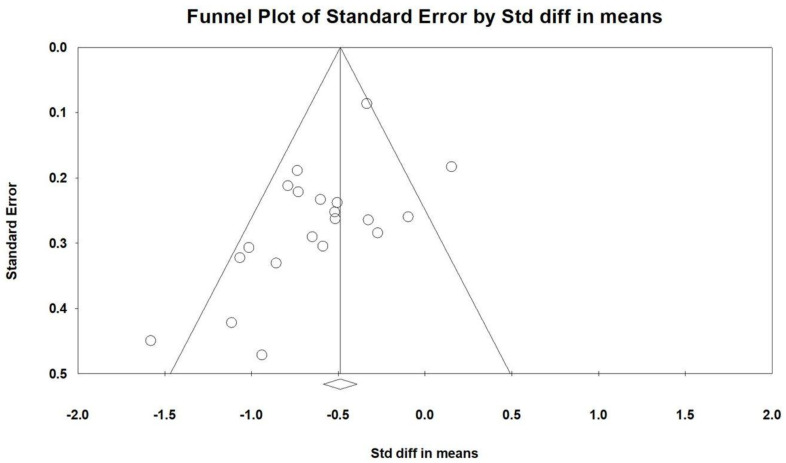
A funnel plot summarizing the findings from all included studies. The diagonal lines represent confidence intervals around the effect estimates, indicating the expected range for the true effect size. Each circle corresponds to an individual study, with larger circles reflecting studies with greater weight or larger sample sizes. The diamond symbol at the bottom represents the overall pooled effect size, with its center indicating the estimated effect and its width denoting the confidence interval.

Egger’s regression analysis detected significant publication bias in the dataset (*p* = 0.00979). Figure 9 presents the funnel plots, depicting the SMD values for the effectiveness of vitamin supplementation. The asymmetry observed in the plot further supports the presence of publication bias. Notably, the visible gap in the lower right corner of the funnel plot raises concerns, suggesting that certain studies with less pronounced effects of vitamin supplementation may be unpublished or undiscovered. This potential absence of data could influence the overall interpretation of the meta-analysis findings.

## 3. Discussion

This updated meta-analysis offers a comprehensive synthesis of the current evidence on vitamin supplementation in the management of chronic pruritus, building upon our previously published work that focused solely on vitamin D [14]. Given the growing interest in micronutrients as adjunct therapies for dermatologic and systemic pruritic conditions, we recognized the importance of re-evaluating the literature to include additional vitamins such as B12, E, and niacinamide (B3). These nutrients have demonstrated promising anti-inflammatory properties and potential for enhancing skin barrier function in recent randomized controlled trials. By expanding the focus beyond vitamin D, this study seeks to reflect the evolving therapeutic landscape and address the current gap in consolidated evidence regarding these alternative vitamin-based interventions.

The findings of this study highlight vitamin B supplementation, particularly vitamin B12, as an effective intervention for reducing pruritus severity. Vitamin B12 demonstrated a pronounced antipruritic effect, especially in patients with atopic dermatitis and chronic kidney disease-associated pruritus. This aligns with previous studies [11,52] suggesting that vitamin B12 modulates neuroinflammatory pathways [56] and supports nerve function, potentially reducing itch perception by stabilizing mast cell activity [57] and inhibiting pro-inflammatory cytokines. Its topical use is further supported by favorable tolerability and ease of application, making it a viable option for long-term symptom management in outpatient settings. Niacinamide (vitamin B3) also exhibited a moderate effect in alleviating pruritus, which may be attributed to its anti-inflammatory, antioxidant, and skin barrier-enhancing properties [17]. Niacinamide has been shown to downregulate nuclear factor-kappa B (NF-κB), a key regulator of inflammation, and reduce oxidative stress, both of which are implicated in chronic pruritus pathophysiology [6]. Furthermore, its role in improving epidermal lipid synthesis and skin hydration suggests a potential benefit for pruritus associated with xerosis or barrier dysfunction disorders [6]. Despite these promising findings, the heterogeneity among studies, particularly in dosage, formulation, and duration of supplementation, remains a limitation. Some trials used oral administration, while others applied topical vitamin B12, which may influence bioavailability and therapeutic outcomes. Future research should focus on standardizing treatment protocols and exploring the long-term efficacy of vitamin B supplementation in different pruritic conditions. Additionally, investigating potential synergistic effects with other therapies—such as moisturizers or antihistamines—may further enhance its clinical utility.

Vitamin E has shown moderate efficacy in reducing chronic pruritus, as demonstrated in our meta-analysis. As a potent antioxidant, vitamin E plays a crucial role in maintaining skin integrity, modulating inflammatory responses, and protecting cells from oxidative damage, all of which contribute to its antipruritic effects. Chronic pruritus is often associated with oxidative stress and increased inflammatory mediators, which disrupt the skin barrier and exacerbate itching sensations. The ability of vitamin E to neutralize free radicals and stabilize cell membranes may explain its observed benefits in itch relief [15]. Several studies have reported the effectiveness of vitamin E in alleviating pruritus associated with dermatological and systemic conditions [10,47]. Its anti-inflammatory action, primarily through inhibition of lipid peroxidation and cytokine modulation, may contribute to reduced skin irritation and hypersensitivity responses [58]. Moreover, vitamin E has been found to enhance keratinocyte function and improve the skin’s moisture retention, which is particularly beneficial in conditions such as xerosis-related pruritus [59]. The administration route of vitamin E also plays a role in its effectiveness. While oral supplementation has been widely studied, topical application may offer more targeted benefits, delivering higher concentrations directly to affected areas and bypassing potential limitations of systemic absorption [60]. This suggests that vitamin E could be an effective adjunctive therapy, particularly for patients with inflammatory skin disorders or pruritus linked to oxidative stress.

Vitamin D has been widely studied for its role in skin health and immune regulation, and our meta-analysis further supports its efficacy in reducing chronic pruritus. The findings demonstrate that vitamin D supplementation, particularly in its D3 form, exerts a moderate antipruritic effect, with topical application yielding greater benefits than oral administration. This aligns with previous research [8,9,42] suggesting that vitamin D modulates inflammatory responses, strengthens the skin barrier, and influences neural pathways involved in itch perception [61]. One potential mechanism underlying vitamin D’s antipruritic effects is its ability to regulate immune function, particularly through its influence on T-cell differentiation and cytokine production [34,62]. Chronic pruritus is often associated with an imbalance between pro-inflammatory and anti-inflammatory cytokines, particularly in conditions such as atopic dermatitis, psoriasis, and chronic kidney disease-associated pruritus. Vitamin D has been shown to suppress the production of pro-inflammatory cytokines such as IL-6, TNF-α, and IL-17 while promoting regulatory T-cell activity, thereby reducing skin inflammation and pruritus intensity [34,62]. Although TNF-α and IL-6 are not considered the primary cytokines in classic pruritus pathways, they are part of the broader inflammatory network involved in various pruritic conditions. Vitamin D has been shown to exert immunomodulatory effects by inhibiting Th1 and Th17 responses and promoting Th2 and regulatory T cell development, thereby shifting the immune balance toward an anti-inflammatory state [63]. This is especially relevant in atopic dermatitis, chronic kidney disease-associated pruritus, and other immune-mediated skin disorders, where dysregulated cytokine profiles contribute to itch. Recent literature also highlights vitamin D’s ability to downregulate IL-17A, TNF-α, and IL-6, which indirectly influences neural and immune crosstalk involved in chronic pruritus [64]. These cytokines can sensitize peripheral sensory nerves or enhance pruritogen release, contributing to the itch–scratch cycle. This study also found that both vitamin D2 and D3 were effective in reducing the expression levels of TNF-α, IL-6, and hs-CRP, further supporting their role in mitigating inflammation-driven pruritus. These findings reinforce the notion that vitamin D supplementation, particularly in its active forms, may serve as a valuable adjunct therapy in chronic pruritus management. However, additional well-designed trials are warranted to determine optimal dosing strategies, treatment duration, and patient populations that would benefit most from vitamin D-based interventions. Our findings support the role of vitamin D, particularly topical D3, as an effective intervention for chronic pruritus. Its ability to modulate immune responses, enhance skin barrier function, and suppress inflammatory mediators underscores its therapeutic potential. Given its favorable safety profile and accessibility, vitamin D supplementation may serve as a supportive option for patients with pruritus related to systemic or dermatologic inflammation.

The administration route of vitamin supplementation plays a crucial role in determining its efficacy in alleviating chronic pruritus. Our findings indicate that topical applications, particularly vitamin B12 and D3, demonstrated superior antipruritic effects compared to oral formulations. This observation may be attributed to the direct cutaneous absorption of topically applied vitamins, allowing for localized anti-inflammatory and neuroprotective effects while bypassing metabolic activation and systemic dilution that can occur with oral administration [16]. In contrast, oral administration of vitamin D2 and D3 exhibited only modest effects in pruritus relief. This may be due to variations in bioavailability and the metabolic conversion process, as vitamin D requires activation in the liver and kidneys before exerting its biological effects [5]. Similarly, oral vitamin B3 and vitamin E demonstrated moderate antipruritic benefits, likely due to their systemic anti-inflammatory and antioxidant properties [12]. However, these effects may take longer to manifest compared to topical formulations, where localized delivery allows for a more immediate response. Additionally, treatment duration appears to influence the effectiveness of vitamin supplementation in pruritus management. Our analysis revealed that short-term interventions (<8 weeks) were associated with greater reductions in pruritus, while longer interventions (>12 weeks) demonstrated diminishing effects. This suggests a potential plateau in efficacy, possibly due to receptor downregulation, physiological adaptation, or declining patient adherence over extended periods [15]. Notably, topical vitamin B12 demonstrated sustained efficacy over 8–24 weeks, while vitamin D3 appeared to exert its most pronounced effects within the first eight weeks of treatment. These findings highlight the importance of considering both the administration route and treatment duration when incorporating vitamin supplementation into pruritus management strategies. Future research should focus on optimizing dosage regimens, comparing different formulations, and investigating potential synergistic effects between various vitamins to maximize therapeutic outcomes.

Despite the encouraging results observed, this meta-analysis has several important limitations that warrant careful consideration. First, the included studies exhibited substantial heterogeneity in terms of study design, participant characteristics, underlying etiologies of pruritus, and outcome evaluation methods. Conditions such as atopic dermatitis, chronic kidney disease, and various inflammatory dermatoses differ in their pathophysiology, which may contribute to variable responses to vitamin supplementation. Although subgroup analyses were performed to account for this variability, residual confounding remains a concern and may limit the generalizability of our conclusions. Future trials should consider adopting more standardized diagnostic criteria and patient selection strategies. Second, evidence of publication bias was detected through funnel plot asymmetry and Egger’s regression test. The underreporting of negative or null findings in the literature could have led to an overestimation of the treatment effect. This highlights the need for journals and researchers to promote the dissemination of all findings, regardless of statistical significance, to ensure a more balanced and accurate synthesis of evidence. Third, inconsistencies in dosage, formulation, and mode of administration among the included trials further complicate interpretation. While topical vitamin D3 and B12 demonstrated relatively stronger antipruritic effects, the dosing regimens—ranging in concentration, frequency, and duration—varied widely across studies. This heterogeneity precludes firm conclusions regarding optimal treatment protocols. Larger, well-designed RCTs are needed to determine dose-response relationships and establish evidence-based guidelines for clinical practice. Fourth, most of the included studies had short follow-up periods, typically ranging from 8 to 12 weeks. The limited duration restricts our understanding of the long-term efficacy and safety of vitamin supplementation in managing chronic pruritus. It remains unclear whether initial improvements are maintained over time or whether physiological tolerance may develop. Longitudinal studies with extended follow-up are essential to address these uncertainties. Fifth, most trials relied on subjective measures such as the Visual Analog Scale (VAS) or Numeric Rating Scale (NRS) to assess pruritus severity. Although these tools are widely used and validated, they are inherently prone to patient-reported bias. The inclusion of objective biomarkers, such as inflammatory cytokines or skin histological changes, in future studies could strengthen the accuracy and reproducibility of outcomes. Sixth, there was a general lack of serum vitamin D level monitoring, particularly in studies evaluating topical formulations. Without tracking systemic vitamin D status, it is difficult to establish a clear relationship between serum levels and clinical improvement. Future research should integrate regular biochemical assessments to clarify this potential correlation and better understand the pharmacodynamics of both topical and systemic vitamin D applications. Lastly, although our meta-analysis focused exclusively on randomized controlled trials to enhance methodological rigor, this approach may have inadvertently excluded relevant real-world data from observational studies and clinical case series. Including such data in future syntheses may provide a more comprehensive view of vitamin efficacy in diverse clinical settings. While this meta-analysis supports the potential role of vitamin supplementation in alleviating chronic pruritus, multiple limitations—including heterogeneity, publication bias, inconsistent dosing, short follow-up, reliance on subjective metrics, lack of serum level monitoring, and exclusion of real-world evidence—should be acknowledged. Addressing these gaps in future research will be critical for refining therapeutic strategies and maximizing clinical benefit.

## 4. Methods and Materials

### 4.1. Data Sources and Selection Criteria

This study conducted a systematic search for RCTs evaluating the effects of vitamin supplementation on chronic pruritus. The search encompassed PubMed, Embase, Cochrane Library, and Web of Science, covering studies published up to January 2025. A structured search strategy was employed, incorporating key terms such as “vitamin”, “chronic pruritus”, “pruritus”, “itch”, and “chronic itch”, with a focus on human clinical trials. The study adhered to the Preferred Reporting Items for Systematic Reviews and Meta-Analyses (PRISMA) guidelines [65], ensuring a transparent and comprehensive review process. Additionally, the reference lists of relevant articles were screened to identify further eligible studies. Studies were excluded if they were case reports, technical papers, conference abstracts, reviews, letters, editorials, or laboratory-based research. This meta-analysis is registered with PROSPERO under the identifier CRD42025642182.

### 4.2. Selection of Studies

The selection of studies was independently carried out by two reviewers, with a third reviewer validating the final decisions to enhance accuracy and minimize bias. To ensure a thorough and in-depth evaluation, full-text versions of all eligible studies were retrieved and carefully examined. A visual representation of the selection process is provided in the PRISMA flow diagram (Figure 1).

### 4.3. Data Extraction

Data extraction was conducted independently using a standardized template, adhering to the methodological guidelines outlined in the Cochrane Handbook [66]. The collected data encompassed key study details, including author names, publication year and country, inclusion criteria, participant demographics (sample size and age distribution), study design, intervention specifics, and outcome assessments. Additionally, the methods used to evaluate chronic pruritus and associated clinical parameters were recorded to ensure consistency across studies.

### 4.4. Outcomes

The main outcome measured was the severity of chronic pruritus, evaluated using validated pruritus assessment tools. Secondary outcomes included alterations in skin lesion area and changes in inflammatory cytokine levels, specifically tumor necrosis factor (TNF), interleukin-6 (IL-6), and high-sensitivity C-reactive protein (hsCRP).

### 4.5. Assessment of Methodological Quality

The quality of the included studies and potential sources of bias were evaluated using the Cochrane Collaboration’s Risk of Bias tool 2.0. Two reviewers independently conducted the assessment, and any disagreements were addressed through discussion with a third reviewer to reach a consensus. Studies were considered to have a high risk of bias if they demonstrated methodological limitations in one or more key areas.

### 4.6. Statistical Analyses

For each included study, the SMD and 95% confidence intervals (CIs) were calculated to compare outcomes between the vitamin supplementation and placebo groups. A random-effects model was applied to account for variability among studies. All statistical analyses were conducted using Comprehensive Meta-Analysis software (version 3.0 Biostat, Englewood, NJ, USA). To assess heterogeneity, the *I*^2^ statistic was used, with values exceeding 50% indicating substantial heterogeneity. Publication bias was evaluated using funnel plots and Egger’s regression test, with statistical significance set at *p* < 0.05 for most analyses, except for publication bias, where a threshold of *p* < 0.10 was applied. Additionally, subgroup analyses were conducted to explore potential sources of heterogeneity, while sensitivity analyses systematically excluded individual studies to assess the robustness of the overall findings.

## 5. Conclusions

This updated meta-analysis highlights the potential of vitamin supplementation, particularly vitamins B12, B3, D, and E, in alleviating chronic pruritus. The findings suggest that topical formulations, especially vitamin B12 and D3, offer superior antipruritic effects compared to oral administration. Shorter treatment durations (8–12 weeks) were associated with more pronounced symptom relief, whereas prolonged interventions did not yield additional benefits. The effectiveness of vitamin supplementation appears to be influenced by factors such as administration route, dosage, and underlying disease conditions. Future well-designed, large-scale RCTs are necessary to establish optimal treatment protocols, refine dosage recommendations, and evaluate long-term efficacy. By integrating vitamin-based interventions into pruritus management strategies, clinicians may offer safer and more effective alternatives for patients suffering from chronic itch conditions.

## Figures and Tables

**Table 1:** Characteristics of included studies.

Author (Year)/Country	Inclusion Criteria	Exclusion Criteria	Sample Size (% of Male)/Age	Study Design	Placebo Using	Intervention/Vitamin Type/Duration	Main Results	Secondary Results
**~8 weeks**								
Ruzicka (2004) [43]/Germany	Patients with scalp psoriasis.	1. Elevated calcium or phosphate levels. 2. Patients who had used topical retinoids, vitamin D3 analogs. 3. Patients with severe comorbidities, known hypersensitivity to vitamin D3 analogs, or conditions that could interfere with the assessment of the study medication’s efficacy, safety, or tolerability. 4. Women of childbearing potential who were not using effective contraception.	P: 273/not explicitly detailed I: 273 (51.2)/average age 45 years	RCT/Double-Blind/Placebo	Placebo emulsion	Tacalcitol emulsion (4 µg/g) applied once daily to the scalp/8 weeks	1. The Tacalcitol group showed a significant improvement in scalp psoriasis compared to the placebo group. The median sum score decreased by 53% in the Tacalcitol group versus 30% in the placebo group (*p* < 0.0001). 2. Tacalcitol was significantly superior to placebo in reducing erythema, scaling, and infiltration.	1. Tacalcitol also showed significant improvement in patient-reported outcomes, including itching and scaling. 2. Treatment was well-tolerated, with similar incidences of local side effects in both the Tacalcitol and placebo groups. No serious adverse events were reported, and calcium homeostasis remained unaffected.
Stücker (2004) [7]/Germany	1. Diagnosed with atopic dermatitis for at least 2 years. 2. Pruritus, typical morphology/distribution, chronic recurrent course, personal or family history of atopic disease. 3. Chronic but not acute inflammatory atopic dermatitis without superinfection.	1. Recent use of topical corticosteroids (last 4 weeks). 2. Recent systemic corticosteroids, ciclosporin, or photopheresis treatment (last 4 weeks). 3. Ultraviolet therapy (last 2 weeks). 4. Participation in another drug trial (last 3 months). 5. Known allergy to vitamin B12 or excipients. 6. Pregnancy or lactation. 7. Strong psychosomatic conditions or poor compliance. 8. Requirement for another topical treatment that could not be discontinued during the trial.	49 participants (30 females, 19 males) Mean age: 33.6 ± 14.1 years	RCT/Double-Blind/Placebo	Placebo cream	Vitamin B12 (cyanocobalamin) topical cream (0.07%) Applied twice daily/8 weeks	Significant reduction in atopic dermatitis severity in the vitamin B12-treated side compared to placebo Modified SASSAD score: Vitamin B12 cream: 55.34 ± 5.74. Placebo: 28.87 ± 4.86. *p* < 0.001 (statistically significant)	Well-tolerated treatment, with no serious adverse events Some mild cutaneous adverse effects (e.g., itching, redness, burning) reported in a few patients.
Kreuter (2006) [42]/Germany	1. Adult patients, at least 18 years old. 2. Continuous intertriginous psoriasis for a minimum of 6 months. 3. Otherwise healthy.	1. Use of systemic corticosteroids, immunosuppressants, UV light therapy (e.g., UV-A, UV-B, psoralen–UV-A) in the previous 4 weeks. 2. Topical treatment of target lesions in the previous 2 weeks. 3. Acute guttate or pustular psoriasis, pregnancy or lactation, severe concurrent infectious diseases, diseases associated with immunosuppression or malignancy, drug dependency, mental dysfunction, or other factors limiting compliance.	P: 20 (60)/53.8 ± 17.1 I: (1% Pimecrolimus): 20 (60)/53.2 ± 14.5 I: (0.005% Calcipotriol): 20 (75)/52.1 ± 13.3 I: (0.1% Betamethaone Valerate): 20 (50)/50.4 ± 11.9	RCT/Double-Blind/Placebo	Placebo cream	1% pimecrolimus, 0.005% calcipotriol, 0.1% betamethasone valerate, or vehicle cream/4 weeks	VAS for pruritus: decreased by 78% for 0.1% betamethasone, 57% for 0.005% calcipotriol, 35% for 1% pimecrolimus, and 43% for vehicle.	1. Mean reduction in MPAS after 28 days: 86.4% for 0.1% betamethasone, 62.4% for 0.005% calcipotriol, 39.7% for 1% pimecrolimus, and 21.1% for vehicle. 2. Betamethasone was significantly more effective than pimecrolimus and vehicle (*p* < 0.05).
Gruber-Wackernagel (2011) [8]/Austria	1. Age above 18 years. 2. Confirmed diagnosis of PLE either by typical patient history and/or histology of skin lesions and/or positive photoprovocation results.	1. Presence of or history of malignant skin tumors. 2. Dysplastic nevus syndrome. 3. Photosensitive diseases such as porphyria, chronic actinic dermatitis, xeroderma pigmentosum or basal cell nevus syndrome. 4. Autoimmune disorders such as lupus erythematosus or dermatomyositis. 5. Psychiatric disorders. 6. Immune deficiency or systemic treatment with steroids and/or other immunosuppressive drugs within 6 months before the study. 7. Pregnancy or lactation. 8. UV exposure in the test fields within 8 weeks before the study. 9. General poor health status. 10. Severe liver or renal disease. 11. Disorders of calcium metabolism or therapy for such disorders with vitamin D-containing drugs.	1. 13 patients (3 men, 10 women; 23% male). 2. Mean age: 37.4 years	RCT/Double-Blind/Placebo/Intraindividual half-body trial	Placebo cream	Calcipotriol cream applied twice daily for 7 days before the start of photoprovocation testing with solar-simulated UV radiation.	Calcipotriol pretreatment significantly reduced PLE symptoms by an average of 32% compared with placebo throughout the observation period from 48 to 144 h after the first photoprovocation exposure (*p* = 0.0022)	Calcipotriol pretreatment resulted in a significantly lower PLE test score in 58% (48 h), 75% (72 h), and 83% (144 h) of the cases compared with placebo Reduced erythema and increased pigmentation were observed with calcipotriol pretreatment
Omidian (2013) [44]/Not mentioned	1. Chronic kidney disease (CKD) patients with refractory uremic pruritus (UP). 2. Must have discontinued all anti-pruritic agents at least two weeks before the study.	Not explicitly mentioned.	Gender distribution and age range not specified in the excerpt.	RCT/Double-Blind/Placebo	Placebo tablets	Oral nicotinamide (vitamin B3) 500 mg twice a day vitamin Type/4 weeks	No significant difference between the reductions in pruritus between groups. However, the interaction effect between drug and time was significant (*p* < 0.026), indicating that the effect of nicotinamide may become more pronounced over time.	All patients completed the study period. The study suggests that a longer intervention period (>4 weeks) might be needed to observe a more significant effect of nicotinamide in reducing uremic pruritus.
Wohlrab (2014) [12]/Germany	1. Female patients aged 18 years or older. 2. Breast cancer. 3. Indicated for adjuvant or neoadjuvant chemotherapy with anthracyclines or taxanes. 4. Combination with trastuzumab was allowed.	1. With pre-existing clinical signs of a barrier dysfunction. 2. History of atopic or psoriatic disposition. 3. Usage of pharmaceutical or over-the-counter products with vasoactive, anti-inflammatory, or diuretic effects. 4. Use of medications affecting lipid metabolism.	95 patients. Age range: 25–77 years	RCT/Not specified as double-blind/reference-controlled crossover study	Usual skincare	Test preparation: a lipophilic cream containing 4% niacinamide (vitamin B3), shea butter, and thermal spring water/6 weeks	1. No significant difference in total DLQI score. 2. Significant improvement in the “Symptoms and Feelings” subscale favoring (*p* < 0.05). 3. VAS scores for pruritus, dryness, and irritability showed significant improvements (*p* < 0.05).	No significant side effects related to the test preparation Patients tolerated the skincare regimen well, with improved quality of life in terms of skin-related symptoms.
Jung (2015) [9]/Korea	1. Patients with chronic kidney disease-associated pruritus (CKD-aP). 2. Patients undergoing hemodialysis.	Not explicitly mentioned in the provided text.	P: 10 (30) I: 10 (30) Mean age: 63.3 years	RCT/Double-Blind/Placebo/Single-center, open-label pilot study	Vehicle solution	Topical vitamin D (calcipotriol) or vehicle solution applied twice daily/4 weeks	1. Both MPAS and VAS scores significantly decreased after 2 and 4 weeks of topical vitamin D treatment compared with the vehicle (*p* < 0.05). 2. Dermoscopic evaluation showed significant improvement in skin dryness for the vitamin D-treated group compared to the vehicle group.	1. No significant side effects were observed. 2. One patient reported a temporary sensation of “feeling heavy” after applying topical vitamin D but continued the treatment without further discomfort.
**8~12 weeks**								
Durakovic (2004) [41]/United States	1. With moderate plaque psoriasis involving at least 5% of body surface area. 2. Two target lesions of at least 5 cm in diameter. 3. Plaque elevation, scaling, and erythema with at least moderate severity (score of 2 on a scale of 0–4).	1. History of hepatic failure, renal failure, nephrocalcinosis, hypercalcemia, hypercalciuria, or hyperphosphatemia. 2. Women of childbearing age who were pregnant, lactating, or unwilling to use effective contraception. 3. Patients using calcium supplements or drugs influencing calcium metabolism.	Paricalcitol-treated Group: 11 (88), mean age 46.5 years (range 29–65).	RCT/Double-Blind/placebo/self-controlled study	Placebo ointment	15 µg/g paricalcitol ointment (19-nor-1α,25-dihydroxyvitamin D2) once daily/12 weeks	Paricalcitol-treated lesions showed a significant decrease in scaling (74%), erythema (69%), and plaque elevation (71%) compared to placebo-treated lesions, which showed reductions of 32%, 22%, and 8%, respectively.	1. Serum calcium and phosphorus levels, as well as the 24-h urinary calcium/creatinine ratio, remained within normal ranges throughout the study. 2. Immunohistochemical analysis showed that paricalcitol treatment markedly reduced the immunoreactivity of transglutaminase K in psoriatic lesions, bringing it closer to the pattern observed in non-lesional skin.
Javanbakht (2011) [10]/Iran	1. Patients diagnosed with AD based on Hanifin and Rajka’s criteria. 2. Objective SCORA between 10 and 70. 3. Normal hepatic and renal function.	1. Use of vitamins, minerals, fatty acid supplements, oral contraceptive pills, steroid hormones (oral or parenteral), anti-epileptic agents, and anticoagulant drugs. 2. Pregnant or nursing. 3. Undergoing phototherapy or taking systemic corticosteroids/immunosuppressive drugs.	P: 11 (10)/26.1 ± 2.8 I: (vitamin D): 12 (33)/21.2 ± 14.6 I: (vitamin E): 11 (37.5)/29.0 ± 2.09 I: (vitamin D+E): 11 (33)/27.5 ± 2.3	RCT/Double-Blind/Placebo	Placebo tablets	Group D: 1600 IU vitamin D3 + vitamin E placebo Group E: 600 IU synthetic all-rac-α-tocopherol (vitamin E) + vitamin D placebo Group DE: 1600 IU vitamin D3 + 600 IU vitamin E/60 days	1. SCORAD scores were significantly reduced in Groups D, E, and DE compared to baseline. 2. Significant reduction in objective SCORAD, lichenification, and pruritus.	1. Plasma α-tocopherol (vitamin E levels) significantly increased in Groups E and DE. 2. No significant adverse effects were reported.
Amestejani (2012) [46]/Not mentioned	1. Diagnosed with AD. 2. Severity of AD evaluated using SCORAD and TIS.	Not explicitly mentioned in the excerpt (potential additional details may be in the full study text)	I: 30 P: 30 Age: Not specified Gender: Not specified	RCT/Double-Blind/Placebo	Placebo tablets	Cholecalciferol (vitamin D3) 1600 IU daily/60 days	1. SCORAD and TIS scores significantly improved in the vitamin D group (*p* < 0.05) 2. No significant improvement in the placebo group (*p* > 0.05) 3. Patients with mild, moderate, and severe AD showed significant improvements with vitamin D supplementation	No adverse effects were reported related to vitamin D supplementation
Shirazian (2013) [45]/United States	1. Adult patients undergoing maintenance HD. 2. Described excessive pruritus. 3. On HD for more than 3 months.	1. PTH level less than 70 pg/mL or greater than 1000 pg/mL. 2. Serum phosphorus greater than 7.0 mg/dL. 3. Serum calcium greater than 11 mg/dL. 4. Active malignancy. 5. Current ergocalciferol treatment.	P: 25 (56)/66.2 ± 13.7 I: 25 (60)/66.1 ± 14.7	RCT/Double-Blind/Placebo/Single-center	Placebo pill	Ergocalciferol 50,000 international units (IU) or placebo once weekly for 12 weeks	Both groups experenced a decrease in pruritus scores, with a reduction of 38.9% in the treatment group and 47.5% in the placebo group. No significant difference in pruritus severity between groups (*p* = 0.34). Adjusted analysis also found no significant treatment effect.	1. No significant differences in calcium, phosphorus, and PTH levels between groups. 2. Significant increase in 25-hydroxy vitamin D levels in the treatment group compared to placebo (19 ng/mL vs. 1.4 ng/mL, *p* < 0.01).
Jaffary (2015) [47]/Iran	Diagnosed with mild-to-moderate AD based on Hanifin and Rajka diagnostic criteria	1. Severe AD with SCORAD > 50 requiring hospitalization. 2. Pregnant or nursing mothers. 3. Coagulopathies or patients using anticoagulant medications 4. Systemic corticosteroid or immunosuppressant users 5. History of allergy to vitamin E	P: 27 I: 28	RCT/Double-Blind/Placebo	Placebo contained no active ingredient and was identical in appearance to vitamin E capsules.	Vitamin E (α-tocopherol), 400 IU daily/16 weeks	1. Significant Itching reduction (*p* < 0.05). 2. Extent of skin lesions reduction (*p* < 0.05). 3. SCORAD index reduction (*p* < 0.05).	1. No adverse effects related to vitamin E supplementation were reported. 2. Women had a greater reduction in pruritus and lesion extent. 3. Men showed a greater decrease in total SCORAD index.
Duca (2017) [11]/Italy	1. Patients with mild-to-moderate plaque psoriasis. 2. Diagnosed based on PASI. 3. Symmetric plaque psoriasis.	1. Other psoriasis forms (palmoplantar, inverse psoriasis). 2. Use of systemic/biological treatments. 3. Allergy to study products. 4. Serious comorbidities affecting study results. 5. Pregnant, breastfeeding, or planning pregnancy. 6. Poor treatment adherence.	1. Total: 24 patients 2. Age: 48.2 ± 15.4 years 3. Gender distribution: 13 males (54.2%), 11 females (45.8%)	RCT/Single-Blind/Intra-patient left vs. right comparison study	The control group used a glycerol-petrolatum-based emollient cream (Cetaphil^®^, Mavena^®^ B12 ointment, Milan, Italy)	1. Topical vitamin B12 (cyanocobalamin) ointment (Mavena^®^ B12) 2. Concentration: 0.07% cyanocobalamin + 20% avocado oil/12 weeks + 4 week washout	1. Significant PASI reduction (*p* < 0.001). 2. Pruritus (itching) and erythema were significantly reduced.	1. After the 4-week washout period, PASI scores slightly increased in both groups, but the vitamin B12-treated side maintained significantly lower PASI scores than the control side. 2. No major adverse effects reported.
Sánchez-Armendáriz (2018) [48]/Mexico	1. Diagnosed with moderate-to-severe AD based on Hanifin–Rajka criteria and SCORAD severity scale. 2. No prior vitamin D supplementation.	1. Primary immunodeficiency disorders. 2. Renal tubular acidosis. 3. Pregnancy. 4. Use of other supplements. 5. Failed to complete the 12-week follow-up.	P: 29/12.2 ± 12.9 I: 29/12.9 ± 10.6	RCT/Double-Blind/Placebo	Received cellulose capsules identical in size and color to the vitamin D3 capsules	Oral vitamin D3 (Cholecalciferol) 5000 IU daily/12 weeks	1. 80% of patients with serum levels < 20 ng/mL still had moderate-to-severe AD, despite treatment. 2. No significant differences in pruritus scores between groups.	No cases of hypercalcemia or other safety concerns were reported.
Mony (2020) [51]/India	1. Adults (20–50 years) with chronic urticaria (CU) for more than 6 weeks. 2. Vitamin D deficiency (serum vitamin D < 20 ng/mL).	1. Acute urticaria, physical urticaria, urticarial vasculitis, hereditary or acquired angioedema. 2. Symptoms of vitamin D deficiency (musculoskeletal pain, fractures). 3. Hepatic or renal dysfunction, malignancies, infections, inflammatory cutaneous disorders. 4. Pregnant and lactating women. 5. Patients who have taken vitamin D supplementation in the past 6 months.	P: 60 (21)/36.71 ± 11.01 I: 60 (20)/38.80 ± 12.54	RCT/Double-Blind/Placebo	Matched placebo	Experimental group: 60,000 IU of vitamin D3 (cholecalciferol) fortnightly for 12 weeks Control group: similar-looking placebo fortnightly for 12 weeks Both groups received standard treatment with levocetirizine	1. Significant reduction in UAS7 scores and medication dosage in the vitamin D treated group compared to the placebo group (*p* < 0.0001). 2. Significant reduction in inflammatory cytokines (IL-6, IL-17, TGF-β, hs-CRP) in the vitamin D treated group compared to the placebo group.	1. Significant increase in 25-OH vitamin D and vitamin D binding protein (VDBP) in the vitamin D treated group compared to the placebo group. 2. No significant change in cytokine concentrations or vitamin D levels in the placebo group.
Mohamed (2022) [50]/Egypt	Adults >18 years of age, having urticaria episodes at least 2 days per week for 6 weeks or longer with/without angioedema	1. With only physical urticaria, urticarial vasculitis, hereditary or acquired angioedema. 2. With dyslipidemia, diabetes, hypertension, pre-existing cardiovascular disease, cerebrovascular accidents, hypothyroidism, smokers, and other systemic or cutaneous disorders including atopic dermatitis, psoriasis, etc. 3. With hypercalcemia (>11 mg/dL), diabetes, renal insufficiency, hepatic disorders, hyperparathyroidism, sarcoidosis, other granulomatous disorders, malignancy. 4. Pregnant and lactating women and patients who have taken vitamin D supplementation in the past 6 months.	P: 67 (50.7)/39.34 ± 7.23 I: 77 (40.2)/36.50 ± 5.12	RCT/Single-Blind/Placebo	Matched placebo	Group 1 (Study group): 38 patients received 0.25 µg alfacalcidol once daily for 12 weeks in addition to the standard therapy (Hydroxyzine 25 mg/day) Group 2 (Placebo group): 39 patients received an oral placebo taken with the same regimen for 12 weeks in addition to the standard therapy (Hydroxyzine 25 mg/day)	The UAS7 total score significantly lower in the study group after active vitamin D administration compared to the placebo group (*p* < 0.01)	1. No significant change in UAS7 total score or number of patients in each severity level in the placebo group compared to their baseline results. 2. Significant increase in mean serum 25(OH) D levels in the study group compared to placebo group and their baseline results (*p* < 0.001). 3. Significant decrease in mean serum IL6, hsCRP, and TNFα levels in the study group compared to placebo group and their baseline results (*p* < 0.01). 4. Significant negative correlation (*r* = −0.67, *p* < 0.05) between serum 25(OH) D levels and total UAS7 scores, indicating disease severity.
**12~24 weeks**								
Nistico (2017) [52]/Italy	1. With a confirmed clinical diagnosis of mild atopic dermatitis (AD). 2. SCORAD index < 25 points.	1. With severe AD or other dermatological conditions. 2. Systemic treatments that could interfere with study results. 3. History of hypersensitivity to the study compounds.	22 Caucasian patients (65)	RCT/single-blind, intra-patient, left-to-right comparative trial.	Comparison was between MB12 cream vs. glycerol-petrolatum-based emollient cream.	Topical vitamin B12 barrier cream vs. glycerol-petrolatum-based emollient cream. 2–3 times per day/12 weeks	1. Both treatments reduced SCORAD scores, but B12-treated sites showed a significantly greater reduction *p* < 0.001. 2. Pruritus severity (VAS scale) reduced from 8.7 to 1.7 in B12-treated sites.	Both treatments were well tolerated.
Disphanurat (2019) [53]/Thailand	1. Diagnosed with chronic plaque-type psoriasis. 2. Mild psoriasis (PASI score < 10)	1. Recent or current use of systemic therapy or phototherapy (within 30 days before enrollment). 2. Hepatic or renal impairment, cancer. 3. Use of immunosuppressive medications, chemotherapy, vitamin D supplements, calcium supplements, bisphosphonates, antiepileptic agents, or anticoagulants. 4. History of hypercalcemia, nephrolithiasis, or parathyroid disease. 5. Pregnant or breastfeeding women.	P: 22 (50)/49.41 ± 15.92 I: 23 (43.5)/52.39 ± 14.19	RCT/Double-Blind/Placebo	Identical-looking placebo pills	1. Vitamin D2 (Calciferol capsules, 20,000 IU per capsule). 2. Three capsules (60,000 IU total) every 2 weeks/6 months.	1. PASI score improvement was significantly greater in the vitamin D group compared to the placebo group at 3 months (*p* = 0.034). 2. PASI percentage improvement (*p* = 0.039).	Vitamin D deficiency significantly reduced in the vitamin D group by the end of the study.
Jenssen (2023) [54]/Norway	1. Active plaque psoriasis (PASI > 0). 2. Baseline serum 25-hydroxyvitamin D [25(OH)D] levels < 24 ng/mL	1. Nut allergy. 2. Primary hyperparathyroidism, granulomatous diseases. 3. Systolic blood pressure > 174 mmHg, diastolic BP > 104 mmHg. 4. Creatinine > 130 μmol/L (men) or >120 μmol/L (women). 5. HbA1c > 9.0%. 6. Pregnancy, history of kidney stones in the last 5 years. 7. Diagnosed or treated for organ cancer or malignant melanoma in the last 12 months. 8. Severe physical or mental illness preventing participation. 9. Use of phototherapy, heliotherapy, or vitamin D supplementation (>800 IU/day). 10. Recent initiation or increase in systemic psoriasis treatment.	P: 61 (63.9)/54.0 ± 9.1 I: 59 (62.7)/53.3 ± 10.9	RCT/Double-Blind/placebo-controlled trial with two parallel groups	Identical-looking placebo capsules	Loading dose: 100,000 IU Followed by: 20,000 IU per week/4 months	1. No significant difference in PASI score changes between the vitamin D and placebo groups. 2. Adjusted difference in PASI: 0.11 (*p* = 0.52). 3. No significant difference in PGA score. 4. No significant difference in self-administered PASI or DLQI scores. 5. No adverse effects reported.	1. Post-intervention 25(OH) D levels increased in the vitamin D group (mean: 29.7 ng/mL) compared to the placebo group (mean: 12.0 ng/mL). 2. Only 41.1% of participants in the vitamin D group achieved 25(OH)D levels > 30 ng/mL.
**>24 weeks**								
Ingram (2018) [49]/New Zealand	1. Adults aged 18 years and older with chronic plaque psoriasis. 2. Patients with stable psoriasis not requiring systemic treatment and with no history of using more than 999 IU/day of vitamin D supplements in the last two months.	1. Chronic kidney or liver disease, smoking, pregnancy, lactation, or planned pregnancy. 2. Use of phototherapy, systemic steroids, or other psoriasis treatments within the last three months.	P: 34 (50)/46.7 ± 13.7 I: 67 (58)/50.7 ± 13.43	RCT/Double-Blind/Placebo	Placebo capsules identical in appearance to vitamin D3 capsules, taken once monthly for 11 months.	Monthly oral doses of 100,000 IU of vitamin D2.	1. No significant difference in PASI scores between the vitamin D and placebo groups over the 12-month period. 2. Both groups showed a mild improvement in PASI scores from baseline, but the improvement was not significantly different between the groups.	Serum 24(OH)D concentrations significantly increased in the treatment group and unexpectedly also increased in the placebo group, possibly due to increased sun exposure or other factors.
Jarrett (2018) [55]/New Zealand	1. Diagnosed with mild psoriasis, confirmed through self-reporting and physician evaluation. 2. Individuals willing to participate in a 12-month follow-up.	1. Current use of vitamin D supplements exceeding 600 IU/day (ages 50–70) or 800 IU/day (ages 71–84). 2. History of psychiatric disorders affecting compliance. 3. Conditions such as hypercalcemia, nephrolithiasis, sarcoidosis, parathyroid disease, or gastric bypass surgery. 4. Participation in another study that could interfere. 5. Baseline serum calcium > 2.50 mmol/L.	P: 42 (65)/64.7 ± 7.4 I: 23 (35)/68.4 ± 8.7	RCT/Double-Blind/Placebo	Sunflower lecithin placebo capsules.	Vitamin D3 (Cholecalciferol) 200,000 IU oral capsule at baseline. 100,000 IU/month/12 months.	No significant difference between vitamin D and placebo groups in psoriasis severity reduction.	Results do not support the use of monthly high-dose vitamin D supplementation for treating mild psoriasis.

AD: atopic dermatitis; DLQI: Dermatology Life Quality Index; HD: hemodialysis; I: intervention; MPAS: modified psoriasis area and severity index; P: placebo; PASI: psoriasis area and severity index; PTH: parathyroid hormone; PLE: polymorphic light eruption; SASSAD: Six Area Six Sign Atopic Dermatitis; SCORAD: SCORing Atopic Dermatitis; TIS: Three-Item Severity Score; UAS7: urticaria activity score over 7 days; VAS: visual analog scale.

## Data Availability

Data included in article.
